# Biomimetic Glycosaminoglycan-Enriched Electrospun Polymeric Scaffolds for Enhanced Early Tissue Regeneration

**DOI:** 10.3390/jfb16120447

**Published:** 2025-11-29

**Authors:** Morgane Meyer, Rana Smaida, Henri Favreau, Cristina Yus, Hervé Gegout, Manuel Arruebo, Nadia Bahlouli, Guy Ladam, Guillaume Conzatti, Stephan Lemmens, Guoqiang Hua, Florence Fioretti, Nadia Benkirane-Jessel

**Affiliations:** 1 Institut National de la Santé et de la Recherche Médicale (INSERM) UMR1260, Nanomédicine Régénérative, 1 Rue Eugène Boeckel, 67000 Strasbourg, France; morgane.meyer2@etu.unistra.fr (M.M.); rana.smaida@lamina.one (R.S.); henri.favreau@chru-strasbourg.fr (H.F.); herve.gegout@unistra.fr (H.G.); conzatti@unistra.fr (G.C.); nadia.jessel@inserm.fr (N.B.-J.); 2 Université de Strasbourg, Faculté de Chirurgie Dentaire, Faculté de Médecine, Faculté de Pharmacie, 4 Rue Blaise Pascal, 67000 Strasbourg, France; nadia.bahlouli@unistra.fr; 3 Lamina Therapeutics, 1 Rue Eugène Boeckel, 67000 Strasbourg, France; stephan.lemmens@lamina.one; 4 Instituto de Nanociencia y Materiales de Aragón (INMA), CSIC-Universidad de Zaragoza, 50009 Zaragoza, Spain; cyargon@unizar.es (C.Y.); arruebom@unizar.es (M.A.); 5 Department of Chemical Engineering, University of Zaragoza, Campus Río Ebro-Edificio I+D, C/ Poeta Mariano Esquillor S/N, 50018 Zaragoza, Spain; 6 PBS (UMR 6270), CNRS, INSA Rouen, UNIROUEN, Normandy University, 55 rue Saint-Germain, 27000 Évreux, France; guy.ladam@univ-rouen.fr

**Keywords:** functional polymeric biomaterial, electrospun scaffold, glycosaminoglycans, cell recruitment, cartilage

## Abstract

Implantable scaffolds are increasingly recognized as transformative tools in regenerative medicine, offering the potential to prevent or mitigate tissue degeneration. Osteoarthritis is a widespread degenerative joint disease that often progresses from early focal lesions to severe joint damage, creating substantial clinical and socioeconomic burdens. Preventive strategies for early-stage lesions remain limited. This study reports the design and development of a functional polymeric scaffold intended to support early tissue regeneration and potentially prevent lesion progression. The scaffold consists of an electrospun poly (ε-caprolactone) nanofibrous membrane enriched with glycosaminoglycans, including hyaluronic acid and chondroitin sulfate, to mimic essential features of the cartilage extracellular matrix and provide a supportive microenvironment. Complete structural, physicochemical, and mechanical characterization was performed to assess the scaffold architecture, stability, hydration properties, and suitability for tissue environments. In vitro investigations were conducted to evaluate cytocompatibility and the interaction of the scaffold with relevant cell types. The scaffold is designed as a potential future preventive strategy to support cartilage integrity and limit disease progression. This approach represents a promising strategy to preserve joint integrity and function, addressing a critical unmet clinical need and enabling translation toward clinical application.

## 1. Introduction

The management of osteoarthritis (OA) remains a significant challenge in the field of healthcare given its high prevalence and impact on individuals’ lives [1]. Despite its widespread occurrence, there is a lack of effective preventive interventions that specifically address the progression of this degenerative disease, creating an urgent and un-met need in the field [2]. Current therapeutic approaches primarily focus on providing symptomatic relief and pain management, while regenerative strategies aimed at halting or reversing cartilage degradation are very limited [3]. Consequently, patients often experience a decline in their quality of life before substantial interventions are initiated [4,5].

Early-stage OA is characterized by minor softening and very fine fibrillation of the cartilage surface on the superficial layer, with minor, localized lesions. These initial changes, often undetectable by standard imaging techniques, mark the onset of cartilage degradation. Clinically, early-stage OA may present with subtle symptoms such as occasional joint pain and stiffness, particularly after periods of inactivity or overuse, but can often be asymptomatic. This stage is critical as it represents a window of opportunity where intervention can potentially delay the progression of degeneration and preserve joint function. Despite the significance of early-stage OA, current treatments are primarily palliative and aim to manage symptoms rather than addressing the underlying disease process.

The treatment protocol for OA management typically begins with recommendations for physical exercise and weight loss. If these conservative measures prove insufficient, the oral administration of analgesics, such as nonsteroidal anti-inflammatory drugs (NSAIDs), is commonly prescribed. As the disease progresses, additional interventions may be considered, including direct injection of NSAIDs into the affected joint [6]. Alternative approaches, such as visco-supplementation with hyaluronic acid, direct stem cell injections, or platelet-rich plasma (PRP) injections, may also be recommended in certain cases, although their long-term efficacy is still being evaluated [7]. However, in cases where OA rapidly progresses, and conservative measures prove insufficient, partial or total joint arthroplasty becomes the last resort. Unfortunately, this invasive and irreversible surgical procedure is unsuitable for younger patients with early OA as it does not offer a stable long-term solution due to the limited lifespan of prostheses and the high likelihood of revision surgeries [8]. Consequently, there is an urgent need to develop effective strategies and therapies specifically targeting the early stages of OA to improve outcomes and delay or prevent the need for surgical interventions.

Between conservative treatments and total joint arthroplasty lies an expanding field of intermediate regenerative medicine approaches aimed at addressing moderate to advanced stages of OA. These approaches aim to restore cartilage function and delay disease progression through both cellular and acellular strategies. Acellular scaffolds, such as collagen-based matrices, synthetic polymer networks, and composite materials, provide structural matrices that support endogenous tissue repair [9,10]. Building on this concept, recent innovations have introduced three-dimensional (3D) biomaterial scaffolds, often derived from animal sources like collagenous membranes such as CaReS^®^ and Chondro-Gide^®^ [11,12]. When implanted on the lesions, these membranes are associated with a process of chondrocyte recruitment. The latest advancements in this field involve directly combining mesenchymal stem cells with these cartilage extracellular matrix (ECM)-mimicking supports [13,14]. However, these collagenous (collagen Type I and III) animal source membranes are not suitable for treating the first stages of OA when placed directly in contact with the cartilage. This is due to their composition that can induce inflammatory responses, which can exacerbate damage rather than facilitate repair; therefore, alternative compositions need to be developed. Inflammation can be detrimental, potentially worsening cartilage damage and accelerating disease progression rather than providing therapeutic benefit. As a result, joint degradation continues until the intervention stage, even though the patient’s quality of life has already been severely impacted [15].

Cell-laden hydrogels represent another promising approach for moderate OA, offering tunable mechanical properties, injectability for minimally invasive delivery, and the ability to encapsulate therapeutic cells within a protective three-dimensional microenvironment [16,17]. These systems, including hydrogels composed of hyaluronic acid, alginate, or gelatin combined with mesenchymal stem cells or chondrocytes, have demonstrated efficacy in promoting chondrogenesis and matrix deposition in preclinical models [18,19]. Functionalization with growth factors or ECM components can further enhance cell differentiation and tissue integration [20,21,22]. Despite these advances, most current regenerative strategies focus on moderate to advanced OA (Grades 2–4), where significant cartilage loss and subchondral bone involvement have already occurred.

Therefore, there is a substantial need for innovative strategies that target the initial phases of cartilage degradation to prevent progression and improve patient outcomes. This unmet need underscores the importance of developing new strategies, such as scaffolds which can provide a proactive and regenerative approach for cartilage preservation.

In order to overcome the aforementioned challenges, this study aimed to develop an innovative polymeric nanofibrous membrane as a potential future preventive therapeutic strategy to slow down OA progression eventually. Our approach involved the utilization of a therapeutic scaffold in order to halt the degradation of articular cartilage before it reaches a critical stage, potentially reducing the need for more extensive interventions in the future. To achieve this, we developed an innovative polymeric fibrous membrane and conducted complete validation of its physicochemical properties, biocompatibility and efficacy. In the field of regenerative medicine, polymers play a crucial role in the development of innovative therapies [23,24,25]. Their diverse properties and tunability offer a versatile platform for designing scaffolds and tissue engineering scaffolds. Biodegradable polymers such as poly (lactic acid) (PLA), poly (lactic-co-glycolic acid) (PLGA), poly (ethylene glycol) (PEG), and poly (ε-caprolactone) (PCL) have been extensively investigated for their potential in cartilage repair and regeneration, particularly in the treatment of knee osteoarthritis [26,27].

### 1.1. Poly (ε-Caprolactone) as a Scaffold Material

In particular, poly (ε-caprolactone) (PCL) was selected for our scaffold due to its unique combination of properties essential for tissue repair.

PCL is a linear, unbranched polyester composed of up to 1000 repeating caprolactone units [28], whose aliphatic structure confers hydrophobicity and solubility in organic solvents such as chloroform, toluene, and benzene, though it remains insoluble in water and alcohol [29,30,31,32]. It is a semi-crystalline polymer with crystallinity inversely related to molecular weight [33], possessing a low glass transition temperature (−60 °C) and melting range (58–64 °C) that enable facile thermal processing but may limit thermal stability, an issue that can be addressed through blending or cross-linking [34,35]. PCL also exhibits excellent thermoformability and shape-memory behavior, making it particularly suitable for advanced fabrication techniques such as 3D printing [36,37,38]. The polymer degrades gradually over 2–3 years, a rate well matched to the timescale of tissue regeneration, while its toughness is enhanced by amorphous domains within its rubbery phase.

PCL exhibits excellent biocompatibility and is FDA-approved for various biomedical applications, ensuring safe long-term contact with tissues [39]. It is biodegradable, providing sustained mechanical support throughout the prolonged tissue regeneration process [27,40,41,42,43,44,45,46]. Moreover, PCL is highly amenable to electrospinning, enabling the fabrication of nanofibrous architectures that closely replicate the native cartilage extracellular matrix (ECM). The resulting nanostructures enhance cell adhesion, migration, and matrix deposition, thereby supporting the restoration of functional cartilage tissue. Therefore, our scaffold consists of an electrospun PCL-based membrane designed to mimic the microenvironment of the cartilage ECM. The use of polymeric PCL fibers provides a large functional surface area and optimal porosity, promoting favorable cell adhesion and proliferation. Together, these attributes make PCL a promising material for the development of biodegradable scaffolds aimed at achieving functional cartilage restoration.

### 1.2. GAG-Enriched Scaffold Relevance

To further enhance the regenerative potential of the polymeric support, glycosaminoglycan (GAG)-type polysaccharides, specifically hyaluronic acid (HA) and chondroitin sulfate (CS) [47,48,49,50], were strategically incorporated into the fibrous membrane based on their essential roles in cartilage homeostasis and protection. CS was selected for its well-established chondroprotective effects: it reduces matrix metalloproteinase (MMP) activity, thereby preventing cartilage degradation, and stimulates proteoglycan synthesis by resident chondrocytes [51,52]. Additionally, CS provides anti-inflammatory properties by inhibiting NF-κB signaling pathways, which is particularly critical in contexts where inflammation can accelerate cartilage breakdown [53,54,55]. HA, the primary GAG in synovial fluid and cartilage ECM, was incorporated for its multifunctional bioactivity: it binds to CD44 receptors on chondrocytes and MSCs to enhance adhesion, chondrogenic signaling, and matrix deposition, while contributing to a hydrated microenvironment that supports cell survival and tissue homeostasis [56,57,58].

Furthermore, both HA and CS serve as bioactive signaling molecules that guide stem cell differentiation toward the chondrogenic lineage [59].

### 1.3. Objective and Novelty: Biomimetic Scaffold Functionality

The synergistic combination of CS and HA within the PCL scaffold is expected to create a biomimetic niche that recapitulates the native cartilage ECM, providing both signaling and structural support to facilitate for endogenous repair [60]. Both HA and CS are key components of the articular cartilage extracellular matrix, and their inclusion allows the supplemented membranes (SM) to closely mimic the physiological microenvironment of native cartilage. Human bone marrow-derived mesenchymal stem cells (hBM-MSCs) were seeded onto these SMs to evaluate their capacity to support adhesion, proliferation, and chondrogenic differentiation, while also assessing the ability of the membrane to recruit surrounding chondrocytes [61,62,63,64].

The primary objective of this study was to develop a multifunctional SM capable of promoting chondrocyte homing at the implantation site, representing a novel preventive strategy for cartilage lesions. These lesions, characterized by very fine superficial fibrillation, are often left untreated due to the absence of specific interventions at this stage. The novelty of our scaffold lies in its dual-function design. First, it combines electrospun PCL fibers with glycosaminoglycans (HA and CS) to create a biomimetic microenvironment that provides simultaneously structural support, bioactive cues, and enhanced surface area to promote cell adhesion and homing, actively recruiting cells on the site of regeneration. Second, the scaffold is designed to be combined with stem cells, enabling accelerated tissue regeneration through synergistic interactions. This dual approach, cell recruitment and stem cell integration, represents a distinctive advancement over existing scaffold designs for tissue regeneration. The SM was designed to intervene for arthroscopic placement to cover the affected area and surrounding healthy cartilage.

Building on these principles, we implemented two complementary experimental strategies. Strategy 1 assessed the ability of GAG-enriched SMs to directly attract and integrate chondrocytes from cartilage-like micromasses placed on the membrane surface, thereby evaluating the intrinsic cell-homing capacity of the scaffold. Strategy 2 extended this approach by embedding hBM-MSC micromasses within a hydrogel layer placed on top of the SM, forming a 3D scaffold–hydrogel–micromass construct. This configuration more closely mimics the native cartilage microenvironment and allows the evaluation of combined cell recruitment, migration, and matrix deposition under physiologically relevant 3D conditions.

Beyond supporting MSC adhesion, proliferation and chondrogenic differentiation, our approach aimed to leverage the functional properties of HA and CS to actively recruit endogenous chondrocytes from the surrounding tissue, a key innovation accelerating in situ cartilage regeneration. Additionally, we explored the use of MSC micromasses embedded within a hydrogel on the SM as a complementary strategy that constitutes another novel feature to further enhance cell homing and matrix deposition, offering a versatile platform for advanced regenerative therapy.

Collectively, these features establish a multifunctional platform that is not only structurally and mechanically robust but also bioactive, representing a first-in-class approach for preventive cartilage repair.

## 2. Materials and Methods

### 2.1. Materials

PCL (Purasorb PC 12, 1.2 dL/g), formic acid, glacial acetic acid, chondroitin sulfate from bovine trachea, 4′,6-diamidino-2-phenylindole DAPI (200 nM), Triton-X100, paraformaldehyde (PFA), and bovine serum albumin (BSA) were obtained from Sigma Aldrich (St. Louis, MO, USA). Hyaluronic acid for the hydrogel (1.5 × 10^6^ Da, 23.7 dL/g) was purchased from Lifecore Biomedical for the. Sodium Alginate (7.5–15 × 10^4^ Da, 20–99 mPa·s^−1^) was purchased from Novamatrix. Cell culture media (Mesenchymal Stem Cell Growth Medium MSC2 and Chondrocyte Growth Medium) were purchased from PromoCell. Calcium chloride solution (10%, 1g/10 mL) was purchased from Renaudin. AlamarBlue^®^ Cell Viability Reagent, Alexa 555 conjugated-phalloidin, Scigen Tissue plus OCT compound cryomatrix, and Alexa 488 fluorophore-conjugated anti-mouse secondary antibody were obtained from Invitrogen Thermofisher Scientific. Trypsin-EDTA, Dulbecco’s Modified Eagle’s Medium phenol red-free culture medium, and hMSC Chondrocyte Differentiation Medium were acquired from Lonza. PBS sterile buffer was purchased from Eurobio Scientific. Hyaluronic acid for the scaffold (1–1.5 × 10^6^ Da) anti-collagen type 2 mouse monoclonal antibody (COL2A1) and anti-aggrecan mouse monoclonal antibody (4F4) was bought from Santa Cruz Biotechnology.

### 2.2. Methods

#### 2.2.1. Membrane Manufacturing by Electrospinning

The aim was to manufacture electrospun polycaprolactone (PCL) membranes as a control (CMs) for the supplemented manufactured electrospun membranes (SMs), 1.2 dL·g^−1^ viscosity polycaprolactone granules were dissolved in a solvent mixture of 97% formic acid (FA) and glacial acetic acid (AA) at a ratio of 70:30 *v*/*v* resulting in a 25% *w*/*v* PCL solution. The solution was stirred using a magnetic stirrer until complete dissolution of the polymer was achieved for 18 h and stored at 2–8 °C, maintaining stability for up to 7 days. For electrospinning, 1 mL of the former solution was loaded into a 2.5 mL syringe and extruded through a 0.5 mm polydimethylsiloxane tube using a syringe pump (ProSense BV) and the Electrospinning Apparatus (EC-DIG, IME Technologies, Eindhoven, Netherlands). The needle-to-collector distance was 20 cm, with an applied voltage of +25/−5 kV and a flow rate of 1 mL·h^−1^. Fibers were collected on aluminum foil and dried at 45 °C under vacuum for 4 h to remove residual solvents.

#### 2.2.2. Manufacture of Supplemented PCL Membranes

The objective was to manufacture supplemented PCL glycosaminoglycan-enriched membranes with enhanced bioactivity. To achieve this, glycosaminoglycans (GAGs), specifically chondroitin sulfate (CS) and hyaluronic acid (HA), were incorporated to provide bioactive cues that mimic the native cartilage extracellular matrix, promote chondrocyte recruitment, and support MSC adhesion, proliferation, and chondrogenic differentiation. Supplemented membranes (SMs) were prepared using the same 25% *w*/*v* PCL solution described for CMs (see Section 2.2.1). As chondroitin sulfate and hyaluronic acid are not fully soluble in the acidic PCL solvent mixture (formic acid/glacial acetic acid), they were first dissolved in ultrapure water: chondroitin sulfate powder at a concentration of 0.4 g·mL^−1^ then hyaluronic acid powder at 0.01 g·mL^−1^ forming an aqueous GAG solution. This solution was then incorporated into the PCL solution and homogenized by agitation. The final solution consisted of 90% polymer and 10% GAGs. The solution was stored at 2–8 °C for up to 7 days and re-homogenized before electrospinning. Electrospinning was performed using the same apparatus and setup as for CMs, with a reduced flow rate of 0.2 mL·h^−1^ due to increased viscosity, maintaining 1 mL per electrospun membrane. Fibers were collected on aluminum foil and dried at 45 °C under vacuum.

#### 2.2.3. PCL Membranes Morphological, Compositional and Stability Characterization

The aim of this set of experiments was to evaluate the physicochemical and structural properties of the membranes, including fiber diameter, thickness, and chemical composition to confirm the incorporation of GAGs. These analyses also served to demonstrate consistency across multiple batches and replicates, validating the manufacturing procedure and reproducibility of composition, with SEM used to prove stability through structural integrity after agitation in Phosphate-Buffer Saline (PBS) and 4 months of storage at room temperature.

Eight-millimeter disks of CMs and glycosaminoglycan-supplemented SMs were mounted on SEM stubs with carbon tape, sputter-coated with gold/palladium (Cressington Sputter Coater 108 Auto, Watford, UK) and imaged by SEM (COXEM EM-30 (COXEM Co, Ltd (Daejeon, South Korea)), and SEM Inspect F50 field emission gun scanning electron microscope (FEI Company, Hillsboro, OR, USA)) to assess their fibrous structure. Fiber diameters were measured from ≥5 SEM images per sample at ×20,000 magnification (n = 200 fibers, 40 fibers per image) using ImageJ 2 2.3.0.

For ultrastructural analysis, scanning transmission electron microscopy (STEM) imaging was carried out using A T20-FEI mission electron microscope (TEM, FEI Company, Eindhoven, The Netherlands) in a probe-corrected Titan (Thermo Fisher, formerly FEI) operated at 300 kV and equipped with a high brightness X-FEG and a spherical aberration Cs-corrector (CEOS). High-angle annular dark field scanning transmission electron microscopy (HAADF-STEM, FEI Company, Eindhoven, The Netherlands) images were obtained with a HAADF detector manufactured by Fischione. Finally, to analyze the chemical composition of the materials, X-ray Energy Dispersive Spectra (EDS) and elemental composition maps were obtained with an Ultim Max detector (Oxford Instruments, Abingdon, UK).

Membrane thickness was measured on cryo-microtome sections (Cryomatrix, −20 °C) imaged under white light (ECHO Revolve, ×4) using ImageJ. Membrane stability was evaluated by re-imaging SMs via SEM after 4 months of storage at room temperature, and measurements were taken as described above to assess fiber and particle integrity.

#### 2.2.4. Membranes Interaction with Aqueous Media

The objective was to evaluate the hydrophilic/hydrophobic properties and water interaction of CMs and SMs, providing insight into the effect of GAG incorporation on membrane water uptake capacity, as increased hydrophilicity can create a adequate environment for cell adhesion and potentially enhance scaffold performance. Hydrophobicity of CMs and SMs was evaluated by contact angle measurements. Three membranes per group were mounted on carbon tape, air-dried for 24 h, and a droplet of ultrapure water was deposited on their surfaces. Contact angles were recorded at deposition and after 10, 20, and 30 min using ImageJ with the “Contact Angle” plugin. Water uptake capacity was determined by weighing membranes in the dry state (*W*0) and after 48 h immersion in 15 mL PBS at room temperature (*W*1) following blotting of excess liquid (n = 3). The swelling was calculated with the following formula:

$$\mathrm{Water}\;\mathrm{uptake}\;\mathrm{capacity}\;=\;\frac{W1-W0}{W0}\times 100$$

#### 2.2.5. Fourier-Transform Infrared Spectroscopy (FTIR) Membranes Analysis

The purpose was to assess the chemical composition of the membranes and confirm the presence of GAGs within the SMs by detecting characteristic polysaccharide functional groups, while also providing evidence of consistent composition across several batches. Raw powders (CS and HA) and CM were evaluated as references to provide a baseline. Infrared spectra of membranes were obtained using a Nicolet 380 spectrometer equipped with an attenuated total reflection (ATR) module (ThermoFisher) at RT. Samples were dried in ethanol, deposited on a diamond ATR crystal, and analyzed directly. Spectra were acquired over 400–4000 cm^−1^ with 32 scans at 1 cm^−1^ resolution and processed using Igor Pro software (Version 6).

#### 2.2.6. Membranes Thermogravimetric Analysis (TGA)

The goal was to evaluate the thermal behavior of the SM and estimate their relative composition, providing further insight into GAG incorporation within the SMs and confirming the reproducibility of material composition across batches. Raw powders (CS and HA) and CM were also evaluated as references to provide a baseline. Thermogravimetric analysis was conducted on previously dried membranes at the “Institut de Science et d’Ingénierie Supramoléculaires de Strasbourg (ISIS)” using a Mettler Toledo TGA/SDTA 851 instrument. The analysis was performed up to a temperature of 1000 °C with a heating rate of 10 °C per hour in air.

#### 2.2.7. Membranes Tensile Testing

The aim of this experiment was to assess the mechanical performance of SMs including stiffness, tensile strength, and elongation at break, to determine how HA and CS incorporation affects the structural integrity and load-bearing capacity of the membranes. Mechanical properties of CMs and SMs were assessed using a universal tensile tester (Instron Dynamight) with a 10 N load cell and Bluehill software (Version V4.42). Three membranes per group were electrospun, dried, and from each, three rectangular templates (10 × 50 mm) were cut. Sample thickness was measured with a digital micrometer (Digimatic Mitutoyo, 0.001 mm resolution) to determine cross-sectional area. Tensile tests were performed at the speed of 10 mm.min^−1^ until failure. The experiment room temperature was set at 17 °C. Young’s modulus (E) was calculated from stress–strain curves as E = σ/ε, while ultimate tensile strength (σ_max) and elongation at break (Eb) were determined for each sample with the respective equations:

σmax = Fmax/a

With Fmax representing the maximum tensile force and being the sample section area,

Eb = ΔL/L × 100%

where ΔL represents the sample elongation at break and L the initial length of the sample.

#### 2.2.8. Cell Studies: Production and Culture

Human bone marrow-derived mesenchymal stem cells (hBM-MSCs) were obtained from donor’s bone marrow and isolated by the Cell Processing Unit of the ECell-France platform at the Centre de Transfusion Sanguine des Armées (CTSA), under the French National Agency for the Safety of Medicines and Health Products (ANSM) authorization. R&D-grade cells were provided for the experiments described below in the context of a grant (ANR ARTITHERA (ANR-19-CE17-0032) between CTSA and INSERM UMR1260). Isolation and expansion followed GMP-compliant protocols, including plastic adhesion, seeding, refeeding, harvesting, cell suspension, and freezing, with quality controls at each step according to the “Guideline on Human Cell-Based Medicinal Products” EMEA/CHMP/410869/2006. For the subsequent experiments, hBM-MSCs at passage 4 were thawed and cultured in T75 flasks with 10 mL MSC2 proliferation medium, refreshed 2–3 times per week until sub-confluency. Cells were detached using trypsin-EDTA after 4 min incubation at 37 °C and counted with trypan blue using a Countess Automated Cell Counter. For three-dimensional (3D) culture, hBM-MSCs were seeded into 96-well ultra-low attachment plates (PrimeSurface^®^, S-Bio, Constantine, MI, USA) at 1 × 10^5^ cells per well and incubated for 72 h to form micromasses for hydrogel embedding and membrane interaction assays. Human articular chondrocytes (passage 6, PromoCell) were thawed and cultured in T75 flasks with 10 mL chondrocyte growth medium, with detachment and counting performed as described for hBM-MSCs.

These cells were used in subsequent experiments to assess the safety and efficacy of the SMs, including cytocompatibility analysis, evaluation of cell adhesion, assessment of chondrogenic differentiation potential, chondrocyte micromass cell-homing on SM surfaces, cell migration assays, and 3D chondrogenic differentiation studies in membrane-hydrogel-based constructs to better mimic the native microenvironment.

#### 2.2.9. Membrane Preparation

Eight mm diameter membrane disks were placed in 48-well plates and sterilized by immersion in ethanol for 1 h, followed by 30 min of UV exposure on each side. Disks were allowed to air-dry completely overnight to ensure full ethanol evaporation prior to further handling for subsequent experiments.

#### 2.2.10. Membrane Cell Seeding

For 2D culture, membrane disks were seeded with 30,000 hBM-MSCs or 30,000 chondrocytes per disk in three biological replicates. hBM-MSCs were maintained in MSC2 proliferation medium for 3 days, then cultured in chondrogenic differentiation medium for 21 days, while chondrocytes were maintained in chondrocyte growth medium. Medium was renewed 2–3 times per week.

#### 2.2.11. Cytocompatibility Analysis: Alamar Blue^®^ Assay

The aim was to assess the cytocompatibility of hBM-MSC-seeded membranes over 21 days to determine whether the SMs support viable, proliferating cells without cytotoxic effects. Cells were evaluated on days 1, 7, 14, and 21 using the Alamar Blue^®^ assay in three experimental replicates. Membranes were washed with sterile PBS and incubated with 10% Alamar Blue^®^ in phenol red-free DMEM at 37 °C and 5% CO_2_ for 24 h. Aliquots (150 µL) were transferred in triplicate to 96-well plates, and absorbance was measured at 570 nm (reduced product) and 595 nm (oxidized product) using a Multiskan FC microplate photometer (Thermo Fisher Scientific). Percentage reduction was calculated according to the manufacturer’s instructions.

#### 2.2.12. Efficacy Assay: Direct Adhesion Assessment of hBM-MSCs on SMs

The objective was to evaluate the ability of SMs to support hBM-MSC adhesion, providing insight into the scaffold’s capacity to promote stable cell attachment, proliferation, and a favorable microenvironment for subsequent tissue repair, single cells were directly seeded onto the membranes and labeled on day 1 by indirect immunofluorescence using phalloidin and DAPI (4′,6-diamidino-2-phenylindole). After the Alamar Blue^®^ assay, the hBM-MSC-seeded membranes were washed three times with PBS and labeled on day 1 through indirect immunofluorescence with phalloidin and DAPI (4′,6-diamidino-2-phenylindole). After the Alamar Blue^®^ assay, the hBM-MSCs seeded membranes were washed with PBS three times. The cells were fixed with 4% PFA for 15 min at 2–8 °C to preserve their structure. Following three additional washes, the hBM-MSCs seeded membranes were incubated with a permeabilizing solution (1% Triton-X100 Sigma Aldrich (St. Louis, MO, USA), PBS) for 25 min, followed by three washes and a 1-hour incubation in a saturating solution (2% bovine serum albumin, BSA) at RT. After three more washes, the hBM-MSCs seeded membranes were labeled with phalloidin conjugated to the fluorophore Alexa Fluor 555 (ThermoFisher, 1/50) for the detection of actin within cells at RT for 1 h. Subsequently, they were washed three times with PBS, and the cell nuclei were labeled with DAPI for 10 min at RT. After three washes with PBS, they were stored in PBS at 2–8 °C until observation. For observation, the hBM-MSCs seeded membranes were placed on a slide without a coverslip or mounting medium and directly examined under an ECHO Revolve hybrid epifluorescence microscope.

#### 2.2.13. Chondrogenic Differentiation Potential of hBM-MSCs Directly Seeded on SMs

The objective was to evaluate whether SMs can support and maintain hBM-MSC differentiation toward the chondrogenic lineage by evaluating the expression of cartilage-specific markers. hBM-MSCs as single cells were directly seeded onto the membranes and cultured for 21 days in chondrogenic differentiation medium. Immunofluorescence analysis was performed to assess the expression of specific markers characteristic of hyaline cartilage, including aggrecan (ACAN) and collagen type II (COL2A1). The hBM-MSCs seeded membranes in chondrogenic differentiation medium were processed as described earlier: washed, fixed, permeabilized, and saturated. They were then incubated overnight at 2–8 °C with primary mouse anti-collagen type 2 (1/200) or anti-aggrecan (1/200) antibodies. After 3 PBS washes, the hBM-MSCs seeded membranes were incubated with Alexa 488 fluorophore-conjugated anti-mouse secondary antibodies (1/200) at RT for 1 h. Nuclei were counterstained with DAPI. The hBM-MSCs seeded membranes were stored in PBS at 2–8 °C and examined under a hybrid epifluorescence microscope as previously described.

#### 2.2.14. Strategy 1: Chondrocyte Micromass Cell-Homing by Integration Directly on SMs Surface

**Scheme 1:**
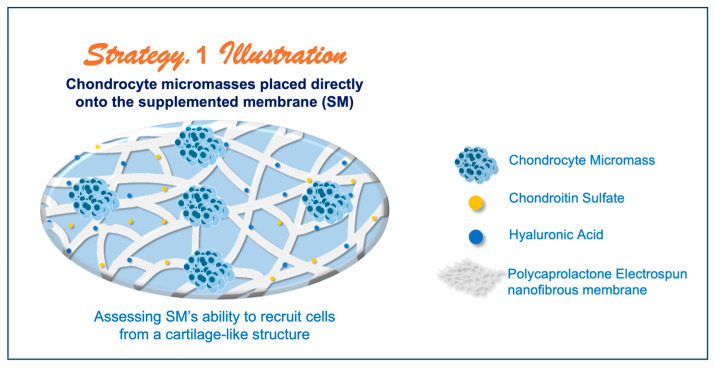
Illustration of Strategy 1.

The objective of Strategy 1 was to evaluate the ability of GAG-enriched SMs to attract, recruit, and integrate surrounding chondrocytes originating from a cartilage-like source (chondrocyte micromasses) assessing whether these cells can infiltrate the nanofibrous network of the SM, thereby mimicking the clinical scenario of implantation in contact with native cartilage in a chondral defect (Scheme 1). Micromasses were chosen here instead of single cells because they provide a physiologically relevant 3D structure that preserves cell–cell interactions and allows us to assess cell migration and integration in a context that better reflects the in vivo native environment [65,66].

Micromasses of chondrocytes (Cell Applications, Inc, San Diego, CA, USA) were generated by depositing 20,000 cells in a 96 U-wells Ultra Low Adhesion (ULA) plate (PrimeSurface^®^ 3D Culture Spheroid plates, S-Bio, Constantine, MI, USA) with 150 μL of chondrocyte growth medium (Cell Applications, Inc). The micromasses were incubated at 37 °C with 5% CO_2_ for 24 h to allow stabilization. Five days after formation, the micromasses were carefully placed directly onto the surface of sterilized SMs and CMs by setting a droplet containing 5 micromasses in the center of the membrane to ensure direct contact. After allowing the micromasses to adhere for 4 h, chondrocyte growth medium was added to the wells. Cell-homing was evaluated at D7 and D14 by fixing the samples with 4% paraformaldehyde, washing with PBS, and staining with DAPI to visualize cell nuclei migrating into the CMs and SMs using fluorescence microscopy. Additionally, at D14, structural interactions between the chondrocytes and membranes were further examined using SEM. Samples were fixed with 2.5% glutaraldehyde, dehydrated in a graded ethanol series, and subjected to critical point drying. Finally, they were sputter-coated with gold and analyzed under SEM to observe cell attachment, migration, and integration into the membrane fibers.

#### 2.2.15. Strategy 2: 3D Assembly of Scaffold–Hydrogel–Micromass Constructs

**Scheme 2:**
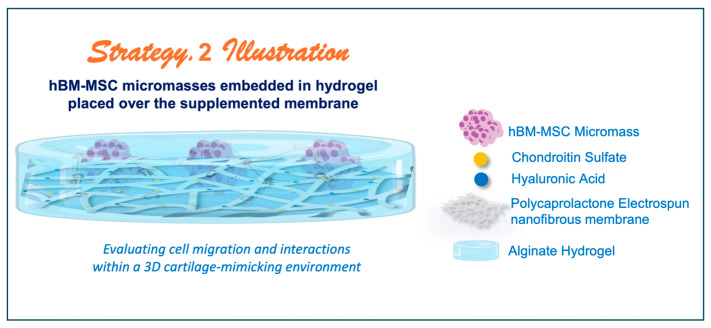
Illustration of Strategy 2.

This second strategy aimed to establish a 3D scaffold–hydrogel–micromass constructs used for subsequent migration and differentiation studies, by embedding hBM-MSC micromasses within a hydrogel layer placed on top of the SM (Scheme 2).

Sterile hydrogel was prepared by dissolving sodium hyaluronate (3 mg·mL^−1^) and sodium alginate (12 mg·mL^−1^) in physiological saline (9 mg·mL^−1^ NaCl). Hyaluronate and alginate were initially reconstituted separately at 37 °C for 5 ± 1 h. Homogeneity was verified by gentle inversion and visual inspection confirming the absence of aggregates as in-process control, with additional mixing or incubation up to 1 h if required. Hyaluronate and alginate solutions were then combined and incubated at 37 °C for 17 ± 7 h, yielding a sterile hyaluronate-alginate hydrogel. Calcium chloride (15 mg·mL^−1^) was prepared separately and used to induce gelation of the hydrogel.

This configuration aimed to form a complete 3D construct, with the micromasses embedded within the hydrogel, to better mimic the native cartilage microenvironment. This approach also investigated the potential of combining SMs with MSC micromasses as a future advanced therapy medicinal product (ATMP) for cartilage repair.

SM disks were placed in 48-well plates and covered with 200 µL of prepared hydrogel. Three hBM-MSC micromasses were carefully placed into each hydrogel to ensure uniform distribution and close contact with the membrane surface. Gelation was induced with calcium chloride, resulting in a solid stable 3D structure suitable for long-term culture. Following gelation, chondrogenic differentiation medium was added, and the constructs were incubated for subsequent migration and chondrogenic differentiation analyses.

#### 2.2.16. Cell Migration Assay and Chondrogenic Differentiation in 3D Constructs

The objective was to evaluate whether embedding hBM-MSC micromasses within a hydrogel that is placed on top of the SM, forming a complete 3D scaffold–hydrogel–micromass construct (with the micromasses embedded within the hydrogel) that better mimics the native microenvironment, could enhance cell recruitment, promote migration into the scaffold, and support chondrogenic differentiation, thereby increasing matrix deposition and accelerating cartilage regeneration. This approach also investigated the potential of combining SMs with MSC micromasses as a future advanced therapy medicinal product (ATMP) for cartilage repair.

The whole construct was fixed with 200 µL of 4% PFA for 15 min at room temperature and washed with PBS. To dissolve the hydrogel and release hBM-MSC micromasses, 300 µL of 15% EDTA was added to each well and gently mixed. Micromasses were collected, fixed in PFA for 15 min, washed, and embedded in freezing resin (OCT) for storage at −20 °C.

For imaging, membranes and released cells that had migrated from the micromasses and reached the membrane surfaces were mounted on glass slides, fixed with PFA, washed with PBS, and nuclei were stained with DAPI for 10 min. Fluorescence microscopy was used to visualize cellular distribution.

To quantify the cells that had migrated from the micromasses into the surrounding hydrogel toward the membrane, the micromasses were first carefully removed, and the membrane was separated to exclude cells that had already migrated onto its surface. The hydrogel containing only the cells that had migrated from the micromasses into the gel, was then dissolved using 15% EDTA to release these cells. The entire hydrogel from each construct was collected and thoroughly homogenized by gentle pipetting to ensure a uniform suspension. The cell suspension was diluted to 1 mL with distilled water, centrifuged at 1000× *g* for 5 min, and the resulting cell pellet was resuspended in 40 µL of distilled water. A 10 µL aliquot of this resuspended sample was used for automated counting with a Countess Automated Cell Counter. Consistent quantification was achieved across samples in replicates at both time points D1 and D21.

hBM-MSC differentiation into chondrocytes was evaluated over 21 days on both membranes and micromasses from the 3D constructs cut into 5 µm slices by indirect immunofluorescence targeting hyaline cartilage markers ACAN and COL2A1. Samples were washed, fixed, permeabilized, and blocked, then incubated overnight at 4 °C with primary antibodies (mouse anti-ACAN or anti-COL2A1). After washing, samples were incubated for 1 h at room temperature with Alexa Fluor 488-conjugated anti-mouse secondary antibodies and 555-conjugated phalloidin. Nuclei were counterstained with DAPI for 10 min. Samples were stored at 4 °C and imaged by fluorescence microscopy.

#### 2.2.17. Statistical Analysis

All experiments and characterizations were conducted on at least three different samples as technical replicates and in three biological replicates. Quantitative values are presented as the mean ± standard deviation of the obtained results. Cytocompatibility replicates were compared using a linear mixed model with two random effects and one fixed effect to identify potential differences. All other data were analyzed using unpaired non-parametric tests for small sample size (Mann–Whitney).

## 3. Results

### 3.1. Structural Characteristics of Membranes

Membranes produced with PCL-CS (PCL–Chondroitin Sulfate) or PCL-HA (PCL–Hyaluronic Acid) were used for comparison with SMs; however, difficulties were encountered during the manufacturing of these membranes. The combination of CS and HA proved to be stabilizing and facilitated the electrospinning process. On the other hand, scaffolds composed solely of PCL-CS or PCL-HA exhibited instability during the electrospinning process resulting in rough surfaces, lacking the desired smoothness. Moreover, their viscosity was not suitable for electrospinning under the same conditions due to the increased viscosity of hyaluronan solutions due to the addition of CS [67]. Therefore, our control group will consist solely of the PCL CMs.

**Figure 1:**
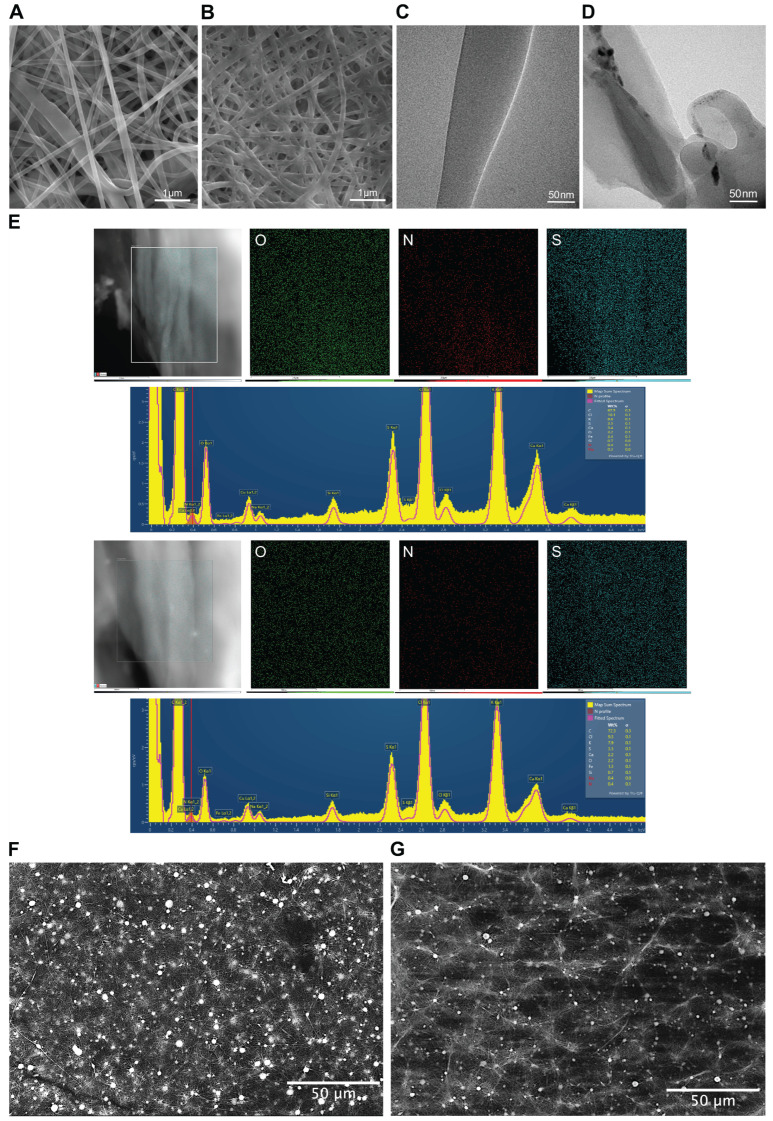
Structural characterization and elemental analysis of CMs and SMs. (**A**,**B**) Representative SEM images showing the surface nanofiber structure of CMs (**A**) and SMs; (**B**) with fibers forming a random, three-dimensional network; (**C**,**D**) HRTEM images illustrating a detailed view of the composing nanofibers of CMs (**C**) and SMs; (**D**) showing spots with higher electron diffraction contrast in the SM nanofibers, consistent with GAG-enriched amorphous domains, where local structural and mass-thickness variations likely account for the contrast differences; (**E**) STEM-EDS microanalysis of the composing elements present in different scanned areas of a SM revealing the presence of N and S corroborating the presence of hyaluronic acid and chondroitin sulfate in the nanofibrous structure. N and S were not detected in the local HRTEM analysis of the CMs (results not shown) The corresponding EDX spectra obtained from the analyzed samples are presented; (**F**) SEM images of SMs after 72 h incubation with continuous agitation at 37 °C, showing no visible deterioration and consistent fiber structure; (**G**) SEM images of SMs after four months of dry storage at room temperature, showing no visible deterioration and consistent fiber structure.

Representative SEM and HRTEM images of the membrane surfaces for CMs and SMs are presented (Figure 1A–D). The average thickness of the CM was 118 μm, while SM had a thickness of 55 μm. The fibers exhibited a random and tangled 3D network structure including also a few polymeric beads. The average fiber diameter for the CM was 163 ± 43 nm, while the average diameter for SM was 130 ± 34 nm. Some spots with high electron diffraction contrast were observed in SMs, but not in CMs probably caused by the incorporation of CS and HA within the nanofibers (Figure 1C,D). These data demonstrate consistent fiber morphology, diameter, and nanostructure across multiple batches and replicates, supporting the reproducibility of the manufacturing process. To further investigate the presence of CS and HA within the membranes, STEM-EDS analyses were performed. As shown in Figure 1E, the elemental composition of different selected areas of the SMs fibers revealed the presence of N and S which corresponds with the incorporation of HA ((C_14_H_21_NO_11_)_n_) and CS (C_13_H_21_NO_15_S) within the supplemented membranes. The N (~0.4 wt.%) and S (~2.3 wt.%) content varied depending on the area selected, which is expected considering that STEM-EDS is a local analytical technique. The superior S content over N can be attributed to the higher weight content of S (7 wt.%) than N (3 wt.%) in CS and the reduced amount of HA included in the SMs compared to that of CS. CM samples did not show the presence of N or S under the same instrumental conditions, further confirming that these elements originate from the supplemented glycosaminoglycans supporting the reproducibility of the membrane’s composition.

In addition, CM and SMs underwent rigorous resistance testing, involving incubation in PBS with continuous agitation (200 rpm) at 37 °C for 72 h. Remarkably, neither structural nor size alteration was observed, indicating the good resilience and durability of these membranes and supporting its stability under these conditions (Figure 1F).

To further assess the stability of the membranes over an extended period, a 4-month storage study was conducted, and their structure was analyzed using SEM (Figure 1G). After dry storage at room temperature, SM maintained its structural integrity, with an average fiber size of 269 ± 26 nm. No visible deterioration was observed, and the membrane remained easy to handle even after 4 months of storage under these conditions, further confirming the stability of the SM.

### 3.2. Infrared Analysis of GAG Presence in Supplemented Membranes

**Figure 2:**
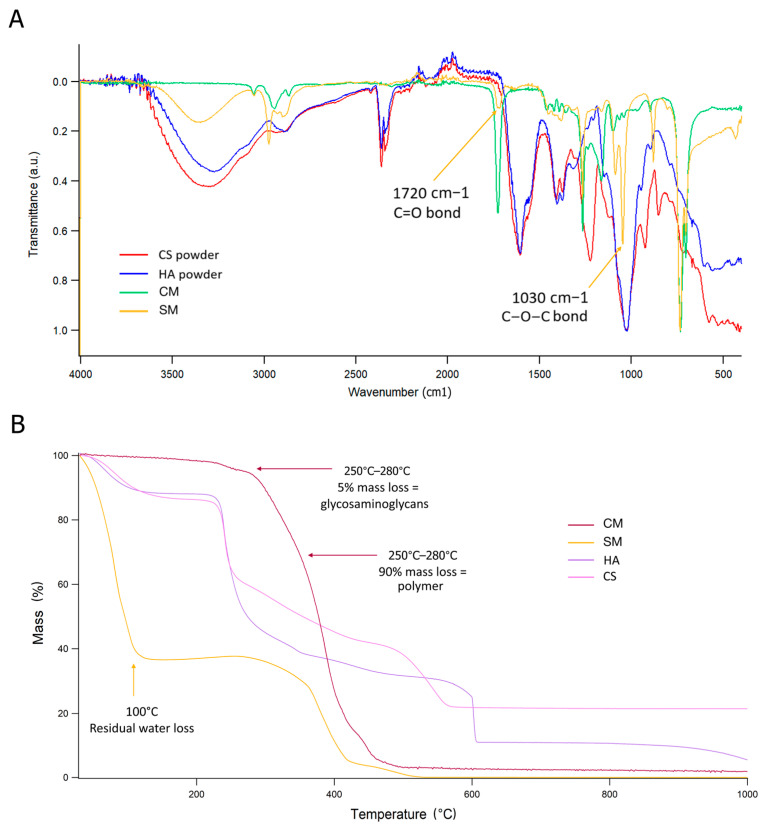
(**A**) FTIR analysis: Spectra of CM, SM, CS and HA raw powders; (**B**) TGA of CM and SM in % of lost weight as a function of temperature.

To further investigate the presence of glycosaminoglycans (GAGs) in the supplemented membranes, an initial infrared spectrum was conducted for SM (Figure 2A, yellow curve), comparing it with the spectra of a CM, hyaluronic acid powder, and chondroitin sulfate powder (green, blue, and red curves, respectively). The broad peak in the range 3500–3000 cm^−1^, corresponding to residual water, was disregarded for composition analysis. The presence of polysaccharides was confirmed by the peak at 1030 cm^−1^, representing the polysaccharide C–O–C skeleton as part of the glycosidic bonds, which exhibited a similar profile to the hyaluronic acid and chondroitin sulfate curves previously reported [68]. The peak at 1720 cm^−1^ in the yellow curve indicated the carbonyl stretch C=O double bond of the ester group in PCL [69], consistent with the peak in the green curve of the CM. However, the distinctive peak at 1150 cm^−1^ in the red chondroitin sulfate powder curve was not observed in the SM spectrum which might be due to a low concentration of chondroitin sulfate in the SM sample. Overall, the infrared spectra confirm the presence of both HA and CS in the supplemented membranes and support the consistent incorporation of these GAGs across batches.

### 3.3. Quantification of Total GAG Mass in Membranes

The thermogravimetric analysis was conducted on SM (Figure 2B), along with CM, and CS and HA powders. The SM exhibited a significant weight loss at temperatures ranging from 300 to 400 °C, which differed from the degradation patterns observed in the control raw materials. The different viscosity in the electrospinning solutions with and without the addition of CS/HA might be responsible for the presence of organic solvent (FA and AA) leftovers in the resulting electrospun fibers. A large amount of organic solvent remained in the fibers of the CM after electrospinning, but all the organic solvent was evaporated in the flight from the needle tip to the collector in the SMs. PCL degradation occurred at approximately 397 °C, while GAG powders showed a slight loss of water followed by degradation at approximately 250 °C. The estimated composition is roughly consistent with the intended PCL/GAG formulation, based on the observed degradation steps in TGA. The observed degradation patterns are consistent with the intended PCL/GAG formulation, further supporting the reproducible incorporation of HA and CS in the supplemented membranes.

### 3.4. Impact of GAG Enrichment on Hydrophobicity

**Figure 3:**
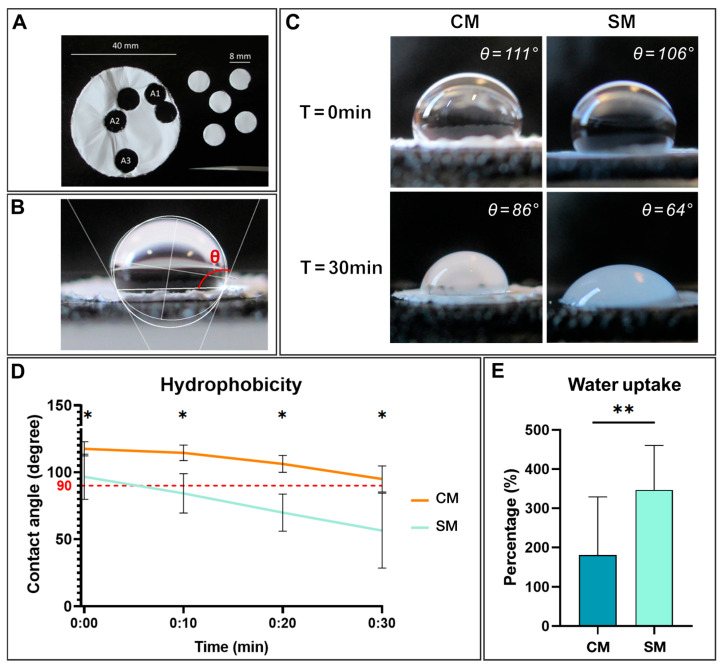
(**A**) Example of disks cut from the membranes in several places (A1 to A3); (**B**) Measurement of the contact angle between an membranes and a water droplet using ImageJ, with the theta angle shown in red; (**C**) Examples of droplets through time; (**D**) Evolution of the contact angle as a function of time CM and SM. (Unilateral Mann–Whitney test at each time point, *: *p* = 0.014); (**E**) Water uptake capacity of CM and SM in *w*/*w* percentage (%), (Unilateral Mann–Whitney, **: *p* = 0.009). Data are represented as mean ± SD.

To evaluate the hydrophobicity of the membranes and its implications, wetting tests (Figure 3B–D) were performed. In the case of the CMs, the contact angle averaged 117 ± 5° during liquid deposition and decreased to 95 ± 10° after 30 min. The contact angle greater than or equal to 90° indicates the hydrophobic nature of the CM [70]. Meanwhile, for SM, the contact angle at deposition averaged 96 ± 17° and decreased to 56 ± 28° after 30 min. Starting between 10 and 20 min, SM angle value stayed below 90° indicating a hydrophilic behavior significantly different from the CM (*p* = 0.014, Unilateral Mann–Whitney).

### 3.5. Water Uptake

To complete the hydrophobicity analysis, the water uptake capacity of the membranes was evaluated (Figure 3E). The average water uptake capacity of SM calculated from nine different samples was 347% *w*/*w*, which is significantly higher than that of CM (180% *w*/*w*, *p* = 0.009, Unilateral Mann–Whitney). The presence of glycosaminoglycans, due to their high polar nature attributed to their large number of hydroxyl, carboxyl, and sulfate groups prone to hydrogen bonding, could be at the origin of this strong increase in water uptake for the SMs because they bind to water and ions [71,72].

### 3.6. Tensile Test

**Figure 4:**
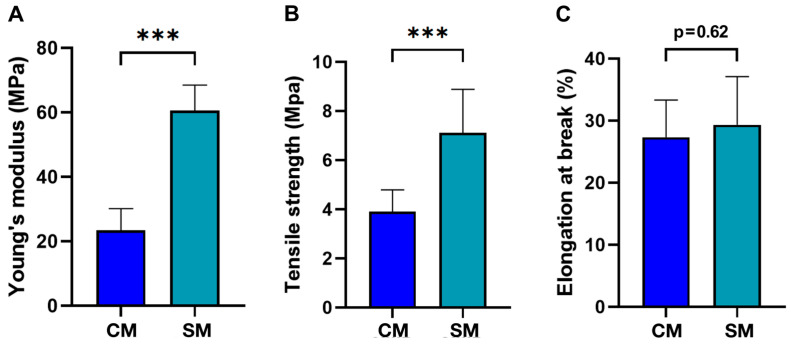
Graph of the membranes Young’s modulus in MPa (**A**), ultimate tensile strength in MPa (**B**) and elongation at break in percentage (**C**) (Unpaired Mann–Whitney test, (**A**) ***: *p* < 0.001) (**B**) ***: *p* < 0.001) (**C**) *p* = 0.62. Data are represented as mean ± SD.

The tensile strength of the membranes was measured by a traction test on nine samples. The Young’s modulus was obtained based on the elastic region of stress vs. strain graphs retrieved from each sample (stress and strain graphs not shown). The mean modulus was 60.6 ± 1.6 MPa for the SM while the CM had a modulus of 23.5 ± 3.8 MPa. Observation of the fibers post-experiment with the SEM showed no structural nor organizational deformation of the fibers. The SM showed significantly higher stiffness (Young’s modulus) than the CM (*p* < 0.001, Figure 4A). Ultimate tensile strength of SMs was also significantly increased (7.1 ± 2.4 MPa) as compared to that of CMs (3.9 ± 0.9 MPa) (*p* < 0.001, Figure 4B). These differences could be attributed to the addition of glycosaminoglycans to the PCL mixture, which may interfere with polymer interactions, as well as to structural variations such as differences in fiber diameters (as previously shown), entanglement differences, or varying porosities. Alternatively, the increase in the mechanical strength of the SMs might be attributed to the load transfer to the CS and HA present in the polymeric blend. No significant difference was observed (*p* = 0.62) for elongation-at-break for the CMs (27%) and SMs (29%) (Figure 4C).

### 3.7. Cytocompatibility

**Figure 5:**
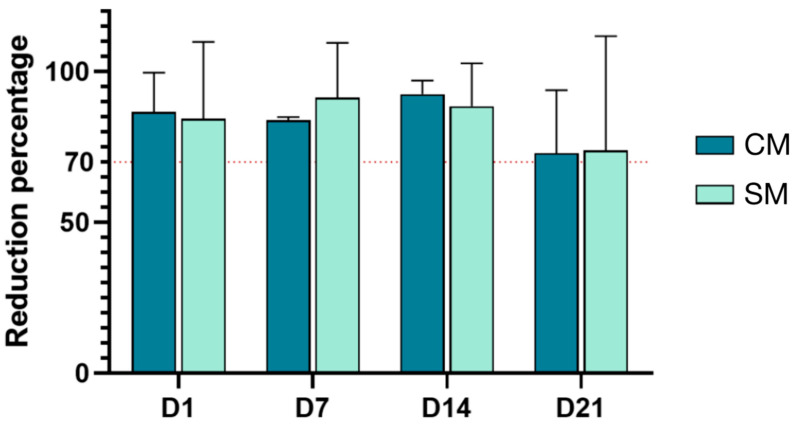
Metabolic activity of hBM-MSCs grown on the CMs and SMs, in a chondrogenic differentiation medium (presented as the percent of reduction of AlamarBlue^®^). Control samples (cells growing on wells lacking membranes) were considered as 100% viability. Data are represented as mean ± SD of at three independent experiments.

During the first week of culture, a substantial reduction rate of resazurin to resorufin was observed, and it exceeded 90% for hBM-MSCs seeded on SM during the second week, demonstrating positive metabolic activity. Although a slight, but not significant decrease in the reduction rate was noted in the third week, the results consistently remained at least 70% of the control rate throughout the experiment for 21 days (Figure 5), confirming the non-cytotoxic nature of our SM in accordance with ISO 10993 standards. Statistical analysis using a mixed linear model revealed that there was no significant difference in the reduction rate between the replicates, indicating the consistency of the results. Furthermore, there was a weak variation in the reduction rate over time (mean of 0.4%, *p* = 0.068). No clear distinction was observed between the CM and SM (1.9%, *p* = 0.62).

### 3.8. Adhesion and Chondrogenic Differentiation Potential

**Figure 6:**
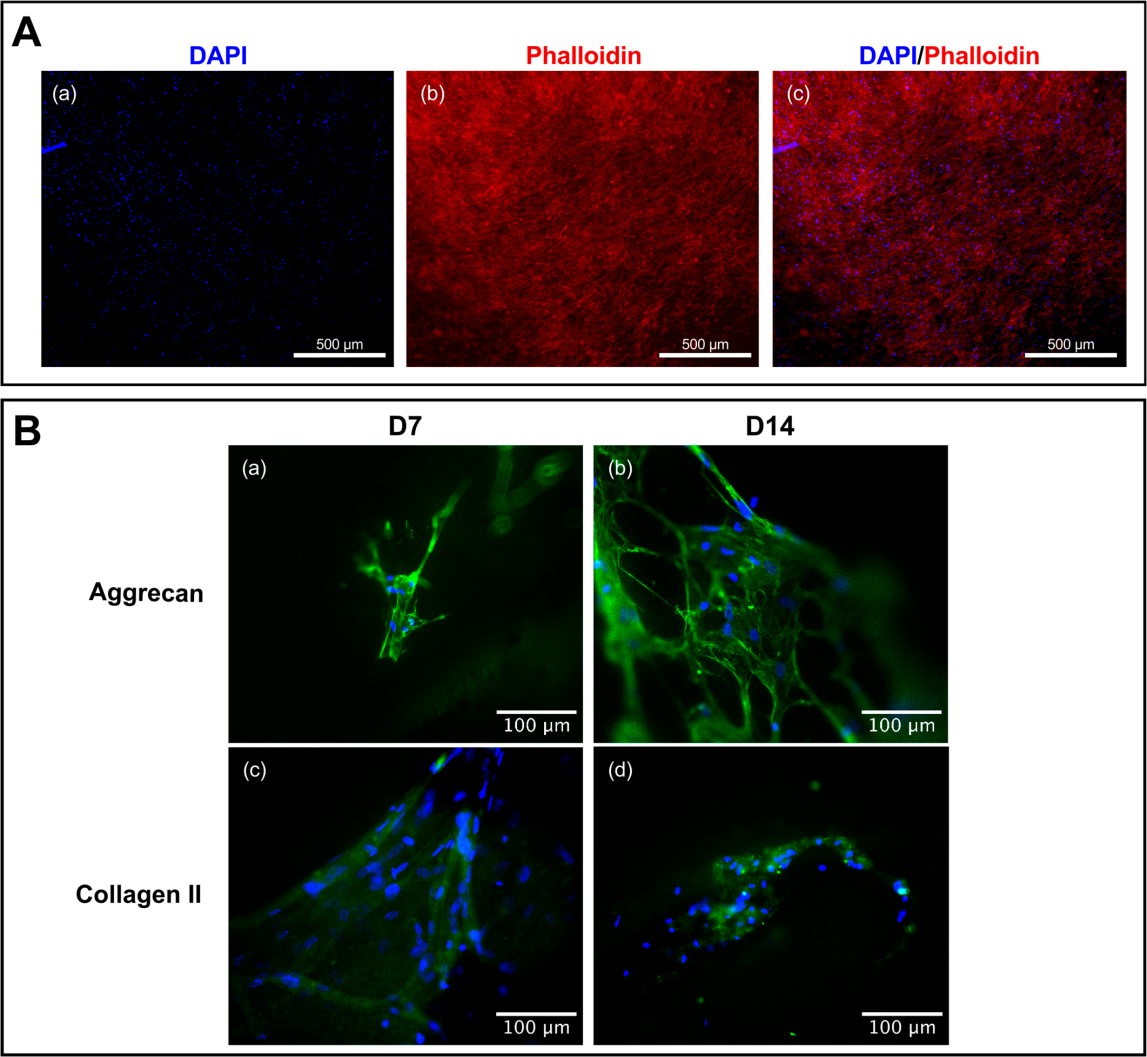
(**A**) Cell adhesion after one week. Indirect immunofluorescence on SM showing DAPI in blue for BM-MSCs nuclei, and Phalloidin in red for cytoskeletal actin; (**B**) Chondrogenic differentiation through indirect immunofluorescence on SM, DAPI (blue) and, aggrecan, collagen type II on days 7 and 14 of culture.

The aim of this experiment was to evaluate the ability of SMs to support hBM-MSC adhesion and chondrogenic differentiation under MSC chondrogenic differentiation medium. Indirect immunofluorescence staining with Phalloidin (cytoskeletal actin, red) and DAPI (nuclei, blue) at day 7 revealed numerous nuclei homogeneously distributed across the SM surface, indicating robust cell adhesion and proliferation (Figure 6A). Phalloidin staining showed well-developed, organized actin filaments and extensive cytoskeletal spreading, confirming strong anchorage of the cells to the scaffold. These observations demonstrate that the surface properties and biochemical composition of the SMs provide a favorable microenvironment for hBM-MSC attachment and maintenance.

Chondrogenic differentiation was assessed in the same MSC chondrogenic differentiation medium by analyzing the expression of cartilage-specific markers: Aggrecan and Collagen type II (green), with DAPI marking cell nuclei (blue), at days 7 and 14 (Figure 6B). Cells on SMs exhibited clear positive staining for both markers at both time points, indicating successful chondrogenic commitment, with this expression maintained consistently throughout the entire 14-day culture period, demonstrating the scaffold’s ability to sustain early chondrogenic differentiation over time. Cells cultured on CMs (data not shown) also expressed these markers due to the differentiation medium, confirming that MSC differentiation is induced under these conditions and that the presence of the membranes do not impair chondrogenic differentiation.

**Figure 7:**
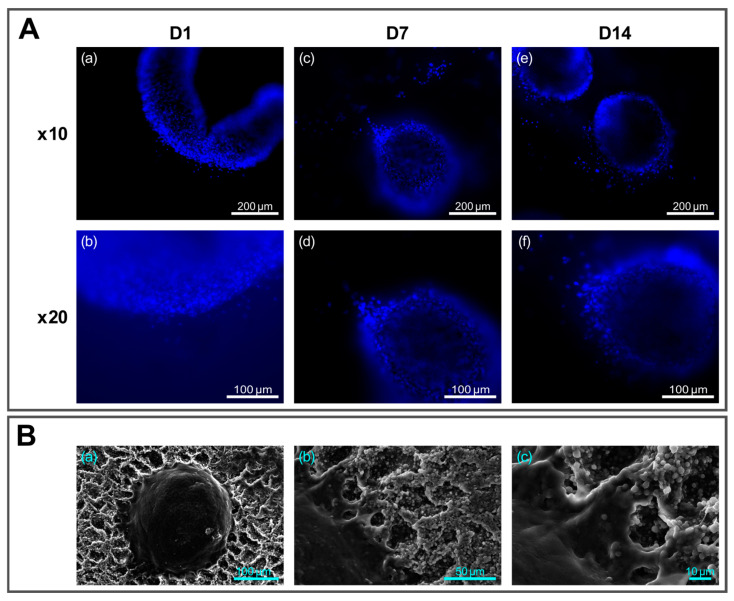
Chondrocyte micromass cell homing and colonization of SMs at D1, D7 and D14. (**A**) Immunofluorescence images (DAPI staining) showing chondrocyte micromasses on SMs at D1, D7 and D14; (**B**) SEM images of chondrocyte micromasses on SMs at D14, providing detailed visualization of cell homing and integration into the membrane fibers.

While both CMs and SMs support MSC differentiation under the differentiation medium, the key question was whether the GAG-enriched SMs can actively recruit surrounding chondrocytes, a property not provided by the CMs. This hypothesis was addressed in the subsequent micromass cell-homing experiment (Figure 7), which evaluated the ability of the SM to attract and integrate chondrocytes, mimicking the clinical scenario of implantation in contact with native cartilage.

### 3.9. Strategy 1: Chondrocyte Micromass Cell-Homing

This Strategy 1 experiment aimed to assess whether chondrocytes originating from a cartilage-like source (micromasses placed directly on the SM surface) can migrate and colonize the SM, mimicking the in vivo situation of a chondral defect in contact with the SM. Immunofluorescence analysis using DAPI staining revealed progressive cell migration from the chondrocyte micromass into the surrounding SM over time (Figure 7A). As early as day 1, initial signs of cell egress from the micromass were visible, confirming the onset of the cell-homing process. By day 7, a continuous migration front of chondrocytes extending along the SM surface was observed. This migration persisted and intensified by day 14, resulting in a higher density of DAPI-positive nuclei distributed within the scaffold structure, indicating active and sustained colonization of the SM.

SEM observations at day 14 corroborated these findings (Figure 7B). The images clearly showed chondrocytes infiltrating and integrating within the nanofibrous network of the SM. Cells exhibited a flattened morphology and close contact with the fibers, suggesting strong adhesion and active remodeling of the scaffold interface. The homogeneous cellular distribution and intimate cell-material interactions confirmed that the incorporation of hyaluronic acid and chondroitin sulfate within the SM matrix provided a conducive environment for chondrocyte attachment, migration, and tissue ingrowth.

### 3.10. Strategy 2: hBM-MSC Micromasses in Hydrogel for Enhanced Cell Recruitment

Based on the results of Strategy 1, which clearly demonstrated that the SM could support cell homing from surrounding chondrocytes, we next tested whether embedding hBM-MSC micromasses within a hydrogel on top of the SM forming a 3D scaffold–hydrogel–micromass construct (Strategy 2), could further enhance this recruitment and accelerate cartilage regeneration. This approach also evaluated the potential of using the SM in combination with MSCs as a breakthrough, highly innovative combined advanced therapy medicinal product (ATMP) to further support cartilage repair.

**Figure 8:**
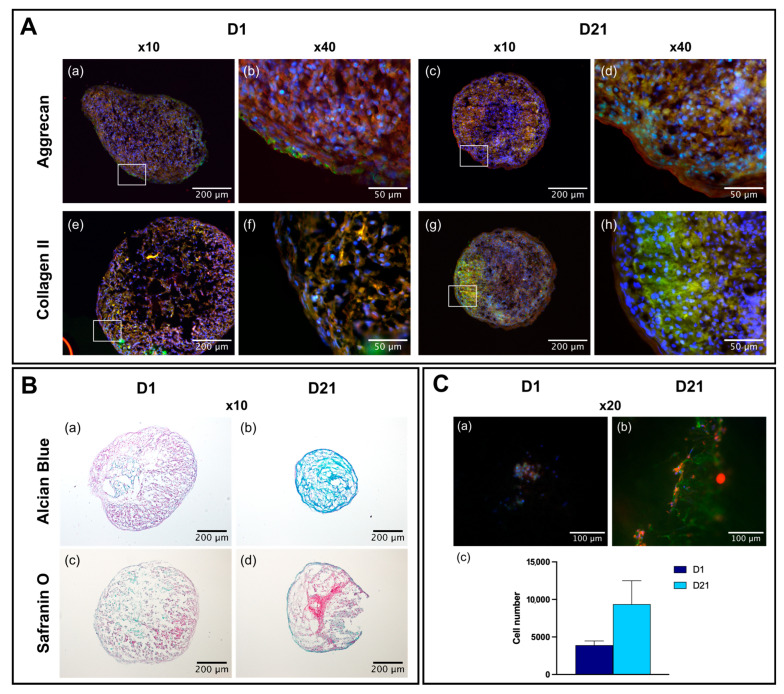
(**A**) Immunofluorescence images of sliced mesenchymal stem cell micromasses cultured in hydrogel on top of the membrane for 21 days, DAPI staining of the nuclei (blue), aggrecan and type II collagen (green) and cytoskeletal actin (red); (**B**) (**a**,**b**) Day 1 and day 21 Alcian blue staining of glycosaminoglycans (blue) with fast red counterstaining on micromasses slices (**c**,**d**) Day 1 and day 21 Safranin O staining of cartilage (pink) with fast green counterstaining; (**C**) Immunofluorescence images of cells from the micromasses adhering to the SM below, (**a**) after 1 day and (**b**) 21 days, marking of aggrecan (green), actin (red) and nuclei (blue), (**c**) number of cells retrieved in the entire hydrogel construct in which the micromasses are embedded at 1 and 21 days.

hBM-MSC micromasses cultured in hydrogel on SMs maintained viability and cytoskeletal organization (Phalloidin, red) and expressed Aggrecan and Collagen type II (green) throughout 21 days of culture, with nuclei marked by DAPI (blue) (Figure 8A). Notably, the strongest Collagen II expression was localized in areas adjacent to the SM, consistent with the zones where micromasses were positioned, while regions further from the SM showed lower staining intensity. Histological analysis showed a similar pattern with Safranin O staining: matrix deposition was mainly localized near the membrane, corresponding to the areas of micromass placement on the SM, whereas more distant zones displayed limited staining (Figure 8B).

Cells migrated from the micromasses into the SM, with few adherent cells at day 1 and substantial colonization by day 21, maintaining Aggrecan expression and actin cytoskeletal organization. Quantification of total cells in the surrounding hydrogel confirmed progressive migration over time. These results indicate that combining MSC micromasses in hydrogel with the SM enhances cell homing and matrix deposition, supporting both endogenous chondrocyte recruitment and MSC-mediated regeneration (Figure 8C).

## 4. Discussion

OA poses a significant challenge within the domain of healthcare, given its pervasive occurrence and profound impact on individuals. Although this disease affects countless lives, the lack of effective preventive measures targeting its progressive nature has given rise to a pressing and unmet demand. Unfortunately, the availability of regenerative treatments to prevent cartilage degeneration remains very limited in today’s therapeutic approaches.

We have previously reported that electrospun polymeric fibers composed of PCL loaded with hydroxyapatite particles or bone morphogenetic protein 2 (BMP-2) could successfully induce apatite formation and MSC differentiation into osteoblasts for bone regeneration [41,42,43,44,45,73]. Building upon this methodology, we present in this study the characterization of manufactured composite scaffolds consisting of HA and CS loaded PCL polymeric fibrous membranes.

The primary aim of this study was to develop an innovative scaffold designed to slow down cartilage degradation at its initial stages, when minor softening and fine fibrillation of the cartilage surface occur, often accompanied by minor, localized lesions. Our scaffold is intended to cover the affected cartilage surface, along with surrounding healthy cartilage, and act as a chondrocyte-homing platform to repair superficial fibrillations. The overarching goal was to evaluate whether the SM, enriched with CS and HA, could provide an optimized structural, biochemical, and mechanical microenvironment to: (i) promote MSC adhesion and chondrogenic differentiation, (ii) facilitate cell homing from surrounding cartilage, and (iii) enable synergistic regenerative strategies using MSC micromasses in hydrogel.

Our study showcased the reproducibility of our manufacturing process in producing SMs with consistent nanostructure and stable characteristics such as fiber diameter and thickness (Figure 1A–D). Notably, the fiber diameter of the SMs was significantly increased compared to the CMs, potentially indicating the incorporation of the desired molecules within the fibers. Transmission electron microscopy confirmed the presence of N and S attributed to HA and CS in the supplemented membranes whereas, those elements were not detected in the control ones (Figure 1E), supporting consistent incorporation of GAGs across batches. In addition, the incorporation of those glycosaminoglycans favored the viscoelastic properties of the solution used during the electrospinning process, resulting in a visually distinct viscous structure of the fibers. Interestingly, the SMs exhibit a thinner profile compared to the CMs, attributed to their larger fiber diameter and a more condensed structure resulting from a reduced distribution rate of the polymer solution during the electrospinning process. Importantly, the integrity and structure of the electrospun scaffolds were preserved following sterilization and after 4 months of storage at room temperature, as verified by SEM (Figure 1F,G), demonstrating stability and durability of the membranes.

FTIR analysis provided evidence supporting the presence of CS and suggesting the presence of HA in the SMs, while these components were absent in the CMs (Figure 2A). To gain deeper insights into the composition of our membranes and confirm consistent composition across batches supporting the reproducibility of GAG incorporation, thermogravimetric analysis (TGA), which involved measuring weight variation as a function of temperature and time, was conducted. As shown in Figure 2B, the TGA results of the SM with CS and HA powders were compared to the CM. During TGA, the SM exhibited a substantial weight loss occurring around the temperature range of 300–400 °C, which differed from the degradation patterns observed in the control raw materials. It is important to note that the observed weight loss in the SM and control samples might be influenced by the presence of residual solvents, potentially affecting the total weight loss measurements. Although the TGA did not provide further insights into the composition of the membranes, it is plausible that the solubilization of the GAGs contributed to a bulk effect, where the cross-linking of the molecules slowed down the degradation process over time. Additionally, interactions between the components may also contribute to this effect on a larger scale, resulting in simultaneous weight losses of the three compounds due to their intertwined nature. The slight shift toward higher temperatures further suggests that the polymers remain stable and that the mixture is well-integrated, consistent with good interactions between PCL and GAGs. Overall, the combined findings from FTIR analysis and TGA provided strong support for the presence of CS and suggested the presence of HA in the SMs, while these components were absent in the CMs, further substantiating the successful incorporation of these molecules into the membranes. STEM-EDS analysis corroborated those hypotheses. The resorbable characteristics of PCL together with the inclusion within the polymeric nanofibers of CS and HA promoted chondrogenic differentiation. The high surface area per volume ratio of electrospun nanofibers would promote biodegradation and the consequent HA and CS sustained release over time. The biological activity of CS and HA was maintained after the electrospinning process promoting cell viability and increasing extracellular matrix synthesis. The elevated water uptake of the supplemented membrane would promote lubrication in the joint favoring motion. Released HA would provide viscoelastic properties to the synovial fluid and CS would stimulate the synthesis of proteoglycans and would inhibit the expression of proteolytic enzymes in chondrocytes. Regarding the storage of these SMs, we stored them at RT based on the knowledge that the components of the scaffold do not undergo any known state transitions within the temperature range of 4 °C to 25 °C. Remarkably, no change was observed in the structure of the membranes after storage at RT for four months, reaffirming their stability and suitability for practical use.

Structural characterization demonstrated that SMs exhibited smooth, homogeneous fibers with reduced diameters compared to CMs (Figure 1A–D). The inclusion of GAGs improved viscoelastic properties during electrospinning, producing a distinct, condensed fiber structure. SMs maintained a thinner profile relative to CMs due to this condensed fiber distribution, while preserving structural integrity following sterilization. The elevated water uptake and hydrophilic surface properties of SMs (Figure 3B–E) created a favorable environment for cell adhesion and protein adsorption, which correlated with the enhanced hBM-MSC adhesion and cytoskeletal organization observed.

Mechanically, SMs exhibited significantly higher Young’s modulus and ultimate tensile strength than CMs (Figure 4A–B), without compromising elongation at break (Figure 4C). These properties supported scaffold integrity during implantation and provided a robust substrate for hBM-MSC adhesion, migration, and subsequent tissue regeneration. Cytocompatibility assays confirmed that hBM-MSC maintained high metabolic activity over 21 days (Figure 5), consistently exceeding 70% of control, further validating the suitability of SMs for cell-based therapies.

Biological evaluation demonstrated that MSCs adhered efficiently to SMs, as evidenced by extensive Phalloidin and DAPI staining (Figure 6A) and maintained a well-organized cytoskeleton necessary for differentiation. Under MSC chondrogenic differentiation medium, cells exhibited sustained expression of Aggrecan and Collagen II over 14 days (Figure 6B), indicating robust chondrogenic commitment maintained throughout the culture period. Beyond supporting MSC differentiation, SMs actively recruited surrounding chondrocytes, as shown in Strategy 1 chondrocyte micromass experiments (Figure 7A–B). In this setup, chondrocyte micromasses were placed directly onto the SM surface to assess the scaffold’s ability to attract and integrate cells from a cartilage-like source. Sequential observations revealed continuous cell migration from the micromass toward the SM, beginning at day 1 and persisting through day 14. This dynamic recruitment closely mimicked the expected clinical response when the SM is implanted in contact with native cartilage, providing an additional mechanism to enhance in situ repair [74].

Further, embedding hBM-MSC micromasses in hydrogel on top of SMs (Strategy 2: 3D Scaffold–Hydrogel–Micromass Constructs) amplified both MSC-driven matrix deposition and endogenous chondrocyte recruitment (Figure 8A–C). Collagen II and Safranin O staining showed matrix deposition localized near the SM, indicating that a more homogeneous distribution of micromasses could improve cartilage regeneration. This approach is particularly relevant for aging patients, who have a limited pool of healthy chondrocytes. Supplementation with MSCs provides functional cells to enhance matrix synthesis and tissue repair, addressing this intrinsic limitation.

Taken together, the SM combined structural integrity, mechanical resilience, and biochemical cues to provide a multifunctional platform for cartilage repair. It supported MSC differentiation, recruited endogenous chondrocytes (Strategy 1), and synergized with hydrogel-based MSCs (Strategy 2) to accelerate regeneration. These properties position the SM as a promising future combined ATMP platform with translational potential for future clinical application in osteochondral repair. The integrated narrative of Figure 1, Figure 2, Figure 3, Figure 4, Figure 5, Figure 6, Figure 7 and Figure 8 illustrated the full continuum from scaffold characterization to MSC differentiation, chondrocyte homing, and hydrogel-assisted regenerative strategies, providing a compelling rationale for future in vivo studies and clinical translation.

## 5. Conclusions

This work established a robust foundation for a new generation of functional electrospun SMs designed to intervene at the earliest stages of OA. By incorporating hyaluronic acid and chondroitin sulfate directly into the PCL nanofibrous matrix, we engineered a multifunctional scaffold that combines mechanical resilience, hydrophilicity, and biological functionality. These properties converged to create a dynamic microenvironment capable of supporting MSC adhesion, proliferation, and chondrogenic differentiation while simultaneously promoting endogenous chondrocyte recruitment, a critical step toward effective in situ cartilage regeneration [74]. The SMs demonstrated reproducibility, cytocompatibility, and stability, retaining their nanostructure and biological efficacy even after storage. Functional assays revealed their ability not only to sustain MSC viability and differentiation but also to actively guide chondrocyte migration and integration, recapitulating the regenerative responses expected in vivo. Together, Strategy 1, with chondrocyte micromasses on the SM, and Strategy 2, with MSC micromasses embedded in hydrogel on top of the SM as a complete construct, demonstrate the scaffold’s dual capacity to recruit cells and enhance matrix deposition, highlighting its potential as an innovative combined ATMP for cartilage repair. Beyond their immediate application, these findings open a new translational horizon for preventive and regenerative treatments in cartilage degradation, where cell recruitment and matrix restoration are still feasible. For aging patients, whose endogenous chondrocyte pools are often depleted, the addition of MSCs provides a means to overcome intrinsic regenerative deficits, restoring tissue functionality and delaying disease progression.

Future work will focus on optimizing micromass distribution, refining hydrogel formulations to achieve homogeneous cellular integration, and validating these results in preclinical in vivo models. Ultimately, this approach holds promise not only for preventing cartilage degeneration but also for establishing a new paradigm in early intervention therapies, where functional biomaterials act as both structural and biological catalysts for joint preservation and regeneration.

## Data Availability

The data supporting the findings of this study are available within the article. Additional data are available from the corresponding author upon reasonable request.

## Abbreviations

The following abbreviations are used in this manuscript:

OA	Osteoarthritis
NSAIDS	Nonsteroid anti-inflammatory drugs
PRP	Platelet-rich plasma
3D	Three-dimensional
ECM	Extracellular matrix
PCL	Poly(ε-caprolactone)
GAGs	Glycosaminoglycans
HA	Hyaluronic acid
CS	Chondroitin sulfate
SM	Supplemented membrane
CM	Control membrane
hBM-MSCs	Human bone marrow-derived mesenchymal stem cells
DAPI	Diamidinophenylindole
PFA	Paraformaldehyde
BSA	Bovine serum albumin
EDTA	Ethylenediaminetetraacetic acid
hMSC	Human mesenchymal stem cells
FA	Formic acid
AA	Acetic acid
SEM	Scanning electron microscopy
STEM	Scanning transmission electron microscopy
FEG	Field emission gun
HAADF	High angular annular dark field
EDS	Energy dispersive spectra
PBS	Phosphate-buffer saline
ATR	Attenuated total reflection
RT	Room temperature
FTIR	Fourier-transform infrared spectroscopy
TGA	Thermogravimetric analysis
ISIS	Institut de Science et d’Ingénierie Supramoléculaire
CTSA	Centre de Transfusion Sanguine des Armées
ANSM	Agence Nationale de Sécurité du Médicament
GMP	Good manufacturing practice
UV	Ultraviolet (radiations)
DMEM	Dulbecco’s modified eagle medium
ACAN	Aggrecan
COL2A1	Type two collagen alpha 1 chain
ULA	Ultra-low adhesion
OCT	Optimal cutting temperature
HRTEM	High-resolution transmission electron microscopy
BMP-2	Bone morphogenetic protein 2
ATMP	Advanced therapy medicinal product
ANR	Agence Nationale de la Recherche
